# Septoplasty Effect on the Enhancement of Airflow Distribution and Particle Deposition in Nasal Cavity: A Numerical Study

**DOI:** 10.3390/healthcare10091702

**Published:** 2022-09-05

**Authors:** Feng Tao, Yu Feng, Baobin Sun, Jianwei Wang, Xiaole Chen, Jiarui Gong

**Affiliations:** 1 Department of Otorhinolaryngology-Head and Neck Surgery, Zhongda Hospital, Southeast University, Nanjing 210009, China; 2 School of Chemical Engineering, Oklahoma State University, Stillwater, OK 74078, USA; 3 School of Energy and Mechanical Engineering, Nanjing Normal University, Nanjing 210042, China

**Keywords:** deviated nasal septum, septoplasty, virtual surgery, computational fluid dynamics (CFD)

## Abstract

The surgery outcomes after fixing nasal airway obstruction (NAO) are sometimes not satisfactory in improving ventilations of airflow. A case study is presented in this paper with computational fluid dynamics applied to determine the key factors for successful septoplasty plans for a patient with a deviated nasal septum. Specifically, airflow, as well as particle transport and deposition were predicted in a pre-surgery nasal cavity model reconstructed from patient-specific Computer Tomography (CT) images and two post-surgery nasal cavity models (i.e., VS1 and VS2) with different virtual surgery plans A and B. Plan A corrected the deviated septal cartilage, the perpendicular plate of the ethmoid bone, vomer, and nasal crest of the maxilla. Plan B further corrected the obstruction in the nasal vestibule and caudal nasal septal deviation based on Plan A. Simulations were performed in the three nose-to-throat airway models to compare the airflow velocity distributions and local particle depositions. Numerical results indicate that the VS2 model has a better improvement in airflow allocation between the two sides than the VS1 model. In addition, the deposition fractions in the VS2 model are lower than that in both the original and VS1 models, up to 25.32%. The better surgical plan (i.e., Plan B) reduces the particle deposition on the convex side, but slightly increases the deposition on the concave side. However, the overall deposition in the nasal cavity is reduced.

## 1. Introduction

Deviation of the nasal septum, i.e., the distortion of the wall between the nasal passages, can result in syndromes such as nasal air passage obstruction [1], epistaxis [2], and headache [3]. Medical researchers concluded that rhinogenic headache and mucous membranes are impacted by airflow caused by nasal septum deviation and related nasal obstruction diseases [4], physical or chemical stimulation of trigeminal nerve [5], mucosal contact [6], and other related factors. Surgeries such as septoplasty are usually needed and executed based on clinical experience, which can hardly predict the airflow distribution changes before and after the planned surgery [7]. Although there are methods that can evaluate the surgery outcomes, e.g., the visual analog scale (VAS) score [8], acoustic rhinometry [9], and nasal resistance tests [10] are applied in certain instances, investigations show that the short-term patient satisfaction rate was between 63% and 88% [11,12]. The unsatisfactory surgery outcomes indicate that it is necessary to optimize the surgery plan using methods that can quantify the airflow distributions in the nasal cavity before and after multiple surgery plans, and the find the best one which can provide the most symmetric flow distributions between the left and right sides of the nasal passage. Rhinomanometers measure the airflow rate and pressure drop when the patient breaths through one nostril. Therefore, they can obtain the ratio of the airflow rate under the same pressure droplet condition between the two nostrils via unilateral measurements [13]. If the left-right nasal cavity flow ratio is smaller than 0.6 or larger than 1.5, it is considered as asymmetric between the two sides, and requires surgery [14].

However, the rhinomanometer is limited in acquiring local airflow distributions in the nasal cavity. Therefore, not so many insights can be generated. To address the deficiency mentioned above, computational fluid dynamics (CFD) based models were used to optimize the surgery plans and evaluate septoplasty outcomes in recent decades. Specifically, Rhee et al. (2011) assessed the nasal resistance, airflow allocation, and distribution in nasal cavity geometries with three different surgical plans, and compared the predicted results with the pre-surgical and post-surgical CFD data [15]. Zhao et al. (2014) suggested that the CFD technique could quantitatively evaluate surgical effectiveness when simulating the virtual middle turbinate resection [16]. Hariri et al. (2015) investigated the pressure drop distribution in different sections of the nasal cavities with different virtual surgical plans for inferior turbinate reduction [17]. Vanhille et al. (2018) claimed that surgeons were positive towards surgery planning using CFD for nasal airway obstruction surgery [18]. However, deposition characteristics of the inhalable particles were not included, which may lead to other complications associated with nasal septum deviation.

Particle deposition in the nasal cavities of healthy subjects has been extensively studied. For example, Kelly et al. (2004) investigated the effect of stereolithography resolution on particle deposition fraction of human nasal airway replicas [19]. Schroeter et al. (2011) studied the impact of surface smoothness of the recreated nasal model on inertial particle deposition [20]. Storey-Bishoff et al. (2008) focused on particle deposition in infant nasal airway replicas [21]. Furthermore, Golshahi et al. (2010) experimentally measured the deposition of ultrafine particles in infant nasal airway replicas [22]. Liu et al. (2010) investigated the air-particle flow in a standardized nasal cavity model based on 30 sets of computed tomography (CT) scans by experiment and simulation [23]. Efforts were also made to investigate the behaviors of nasal spray, inhalers [24], and other medical devices [25,26] used for disease treatments in the nasal cavity. Except for spherical particles, the deposition of ellipsoidal fibers in the nasal cavity also attracted attention [27,28,29], due to their different aerodynamic behaviors than spheres.

Inhalation of particulate air pollution is associated with sinusitis [30,31] and allergic rhinitis [32]. Based on clinical observation, there is a possible causal relationship between septal deviation and sinus disease [33]. Kucybała et al. (2017) analyzed 214 patients and concluded that nasal septal deviation is relevant to the development of maxillary sinusitis [34]. Yousem et al. (1991) indicated a statistically significant relation between nasal septal deviation and sinusitis [35]. From the fluid-particle dynamics aspect, Inthavong (2019) indicated that a smaller curvature ratio leads to stronger secondary flow motion, which enhances particle deposition [36]. The concave and convex surfaces of the deviated septum decrease the local curvature ratio. Therefore, the deviated septum can enhance the deposition of inhalable particles in the nasal airway, which may cause sinusitis and allergic rhinitis. However, the transport dynamics and deposition patterns of inhaled particles in the diseased nasal cavity are still unknown.

**Figure 1:**
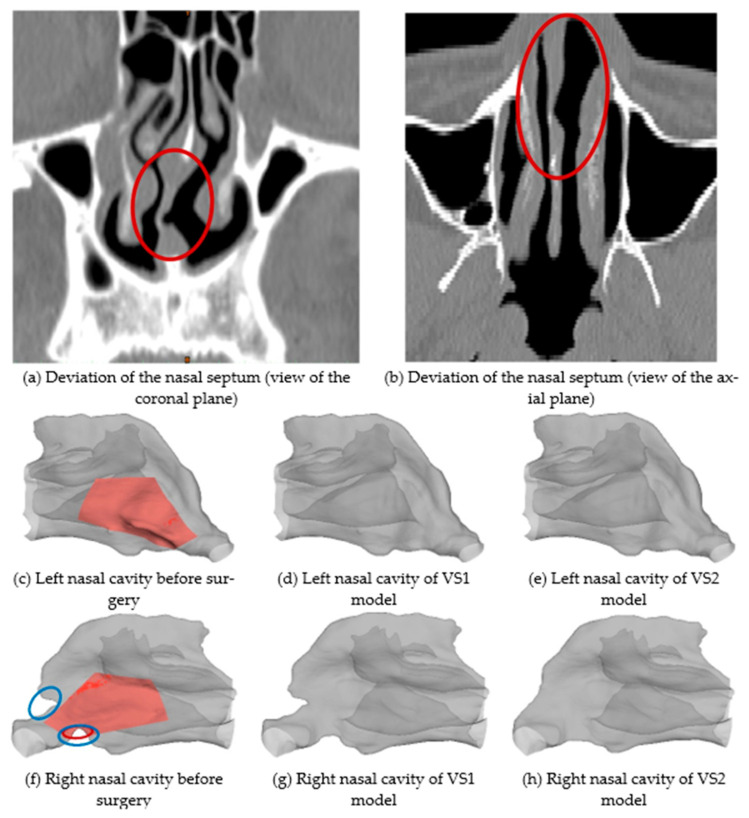
Details of the nasal cavity geometries employed in this study: (**a**) deviation of the nasal septum (view of the coronal plan), (**b**) deviation of the nasal septum (view of the axial plan), (**c**) pre-surgical geometry of the left nasal cavity, (**d**) post-surgical geometry of the left nasal cavity after VS1, (**e**) post-surgical geometry of the left nasal cavity after VS2, (**f**) pre-surgical geometry of the right nasal cavity, (**g**) post-surgical geometry of the right nasal cavity after VS1, and (**h**) post-surgical geometry of the right nasal cavity after VS2.

This paper serves as a case study to evaluate the surgical outcomes with different plans via the comparisons between the airflow and particle deposition patterns. Specifically, based on the spiral CT scan data of the head region of a patient with nasal septum deviation (see Figure 1), the 3D pre-surgical nose-to-throat geometry was reconstructed. In accordance with the two virtual surgery plans proposed, the pre-surgery geometry was modified to represent two post-surgery geometries, respectively (see Figure 1). Simulations of airflow and deposition of inhalable particles for these airway models before and after virtual surgeries (i.e., the original model, VS1 model, and VS2 model) were performed. VS1 model is the result of the surgical plan executed (i.e., Plan A), in which the patient claimed no noticeable improvement. VS2 model is created based on an optimized virtual surgical plan (i.e., Plan B). The surgery outcomes in this study were evaluated by comparing flow characteristics, including airflow allocation, airflow velocity distribution, and particle deposition characteristics, including deposition fraction (DF) and deposition pattern.

## 2. Methodology

### 2.1. Nasal Cavity Geometries and Meshes

Figure 1a,b are the coronal and axial views of the spiral CT results obtained from a mid-age male patient (age of 41, weight of 69 kg, BMI of 23.9 kg/m^2,^ and ASA Class 1) with a deviated nasal septum. Based on the spiral CT scan data, the reconstruction of the nose-to-throat respiratory tract was conducted, and two surgical plans were proposed. The nasal cavities shown in Figure 1c–h were visualized from the position of the septum to illustrate the changes in geometry due to different surgical plans. Figure 1c,f show the left and right sides of the pre-surgical nasal cavity geometry, respectively.

**Figure 2:**
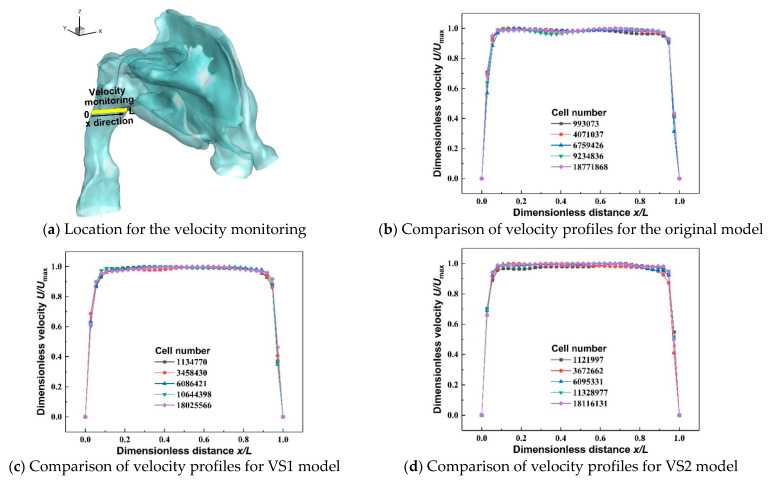

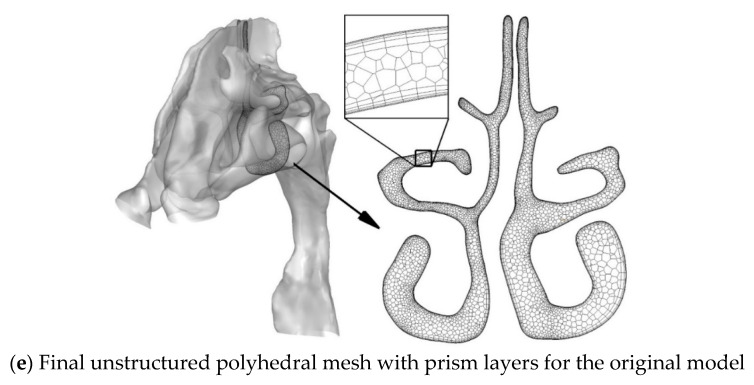
Mesh independence tests and mesh structure.

The first virtual surgery (VS) plan, i.e., Plan A or VS1, which is the actual surgery performed on the patient. VS1 resected partial septal cartilage, the vertical plate of the ethmoid bone, and vomer (see highlighted red regions in Figure 1c,f). It also partially repaired the maxillary nasal ridge (circled red in Figure 1f). The two sides of the nasal cavity of the VS1 model, i.e., the results after surgery using Plan A, are shown in Figure 1d,g. However, the patient claimed that breathing improvement after the surgery was not significant. Accordingly to the feedback, another virtual surgery plan, i.e., Plan B or VS2, was designed. Specifically, Plan B further corrected the obstruction in the nasal vestibule and caudal nasal septal deviation based on Plan A, which is circled blue in Figure 1f. The two sides of the nasal cavity of the VS2 model after surgery performed after Plan B are shown in Figure 1e,h. The whole geometry of the original nose-to-throat airway is shown in Figure 2. The nasopharynx, laryngopharynx, and throat remained the same for VS1 and VS2 models. The area of the virtual surgeries does not include the maxillary sinus. Indeed, the airflow that enters the maxillary sinus is not significant and does not influence the breathing feelings very much. Therefore, the maxillary sinus was not reconstructed in this study. However, if the size of the maxillary sinus may change and affect the cross-sectional area of the nasal cavity [37], the maxillary sinus needs to be included. 

Unstructured polyhedral meshes with prism layers for the three nasal cavity models were generated, and mesh independence tests were performed (see Figure 2). Specifically, the yellow line shown in Figure 2a illustrates the location for the velocity monitoring. For each geometry of the nasal cavity, five meshes with different total cell numbers were generated to compare the dimensionless velocity profile variations in the pharynx region with different mesh densities. Considering the differences in dimensionless velocity profiles between the 6.75 million and 18.77 million meshes in Figure 2b are all smaller than 5%, the mesh with 6.75 million cells is selected as the final mesh. Similarly, the final meshes for the VS1 model and VS2 model contain 6.09 million cells and 6.10 million cells, respectively. The details of the final mesh for the original model are shown in Figure 2e as an example.

### 2.2. Governing Equations

#### 2.2.1. Continuous Airflow Phase

To evaluate the surgical effect on the airflow distribution in the nasal cavity, two inhalation airflow rates (i.e., 15 L/min and 60 L/min) were employed to cover both laminar and turbulence flow regimes that can exist. Specifically, laminar flow occurs at a low inhalation flow rate, say, 15 L/min, in the nasal airway. The average inlet Reynolds number at the nostrils is approximately 970. Therefore, the governing equations for the laminar flow are

(1) $$\nabla \cdot \vec{u}=0$$

(2) $$\frac{\partial \vec{u}}{\partial t}+(\vec{u}\cdot \nabla )\vec{u}=-\frac{\nabla p}{\rho }+\nabla \cdot [\upsilon (\nabla \vec{u}+{\left(\nabla \vec{u}\right)}^{tr})]$$

Turbulent flow occurs at higher flow rates, i.e., 60 L/min. Previous studies have shown that the shear stress transport (SST) model combined with the eddy interaction model (EIM) can accurately predict the deposition fraction of inhalable particles in the nasal airway in a wide range of $d_{a}^{2}Q$ [38]. The transition SST model is improved based on the SST model to capture the flow transition and separation phenomena [39,40]. Experimental results showed that flow separation existed in the pharynx region [41]. Therefore, in this paper, the airflow in the nose-to-throat airway at the inhalation flow rate of 60 L/min was simulated by the transition SST method. More details of the following governing equations can be found in existing literature [39,40,42].

(3) $$\frac{\partial }{\partial t}\left(\rho k\right)+\frac{\partial }{\partial x_{j}}\left(\rho u_{j}k\right)={\tilde{P}}_{k}-{\tilde{D}}_{k}+\frac{\partial }{\partial x_{j}}\left(\left(\mu +\frac{\mu_{t}}{\sigma_{k}}\right)\frac{\partial k}{\partial x_{j}}\right)$$

(4) $$\frac{\partial }{\partial t}\left(\rho \omega \right)+\frac{\partial }{\partial x_{j}}\left(\rho u_{j}\omega \right)=\alpha \frac{P_{k}}{\nu_{t}}-D_{\omega }+Cd_{\omega }+\frac{\partial }{\partial x_{j}}\left(\left(\mu +\frac{\mu_{t}}{\sigma_{\omega }}\right)\frac{\partial \omega }{\partial x_{j}}\right)$$

(5) $$\frac{\partial }{\partial t}\left(\rho \gamma \right)+\frac{\partial }{\partial x_{j}}\left(\rho u_{j}\gamma \right)=P_{\gamma 1}-E_{\gamma 1}+P_{\gamma 2}-E_{\gamma 2}+\frac{\partial }{\partial x_{j}}\left(\left(\mu +\frac{\mu_{t}}{\sigma_{\gamma }}\right)\frac{\partial \gamma }{\partial x_{j}}\right)$$

(6) $$\frac{\partial }{\partial t}\left(\rho {\tilde{Re}}_{\theta t}\right)+\frac{\partial }{\partial x_{j}}\left(\rho u_{j}{\tilde{Re}}_{\theta t}\right)=P_{\theta t}+\frac{\partial }{\partial x_{j}}\left(\sigma_{\theta t}\left(\mu +\mu_{t}\right)\frac{\partial {\tilde{Re}}_{\theta t}}{\partial x_{j}}\right)$$

The definitions of the symbols and variables are listed in Nomenclature.

#### 2.2.2. Discrete Particle Phase

The movement and deposition of micron particles (2 μm to 10 μm in diameter) in the upper respiratory tract are mainly affected by drag force and gravity, and therefore, the governing equation of inhalable particles is [42,43]

(7) $$m_{p}\frac{d{\vec{u}}_{p}}{dt}=\frac{1}{8}\pi \rho d_{p}^{2}C_{Dd}\left(\vec{u}-{\vec{u}}_{p}\right)\left|\vec{u}-{\vec{u}}_{p}\right|+m_{p}\vec{g}$$

The EIM model [38,42,44] is achieved using the in-house user-defined functions (UDFs), which recover the influence of random vortex on the particle motion in the turbulence.

### 2.3. Numerical Setup

To investigate the effect of the virtual surgeries on the flow distribution and deposition of inhalable particles, two inhalation flow rates, i.e., 15 L/min and 60 L/min, were examined. Environmental pressure (i.e., zero gauge pressure) was applied at the inlets, and the throat outlet had negative gauge pressure to mimic the inspiration flow caused by lung expansion. The values of the negative gauge pressure of the outlet were adjusted for different nasal airway models to ensure the same inhalation flow rate.

Considering that the inhalable particles are dilute phase, one-way coupling was applied. After the convergence of the continuous phase simulation, 10,000 particles with a density of 2650 kg/m^3^ were released randomly at the entrance of the nasal cavity. The positions of the deposition of particles in the nasal cavity, nasopharynx, laryngopharynx, and throat were recorded separately, as well as the particles escaped from the throat outlet. The regional deposition fractions and deposition patterns of particles with different diameters, i.e., 2, 4, 6, 8, and 10 μm, were compared for the three nasal airway models. The effect of the particle number on the regional deposition fraction was tested. Adding particle numbers to 15,000 or 20,000 only affected less than 1.0% of the regional deposition fraction.

### 2.4. Validation of Model

Particle deposition under the laminar flow condition has been validated in our previous study [43]. Zhang and Kleinstreuer (2011) indicated that the transition SST model is suitable for turbulent airflow simulation in human airways. [45] The predicted particle deposition fraction with the UDF-enhanced discrete phase model (DPM) and the transition SST model have been compared with experimental data [42]. The most significant deposition fraction discrepancy between our predictions and experimental results was less than 12%, with inhalation flow rate *Q*_in_ = 30 L/min and 90 L/min.

## 3. Results and Discussion

### 3.1. Airflow Characteristics

Table 1 shows the airflow allocations in left and right nasal cavities for the three nose-to-throat models under rest and moderate exercises. Before the surgery, there are significant differences in the flow rates of the left and right sides of the nasal cavity due to the deviation of the nasal septum. For both inspiratory intensity conditions, there are only less than 17% of the airflow passed through the right side.

The airflow allocations of the left side increased by 6.1% to 8.0% for the VS1 model at 15 L/min and 60 L/min, respectively. It suggests that surgical plan A has a limited effect on correcting the unbalanced flow distribution. For the VS2 model, the inhalation flow rates are doubled based on the flow rates of the VS1 model, demonstrating a significant improvement. It is worth mentioning that nasal cycling may also contribute to the higher inhalation flow rate in the left nasal cavity [15].

**Figure 3:**
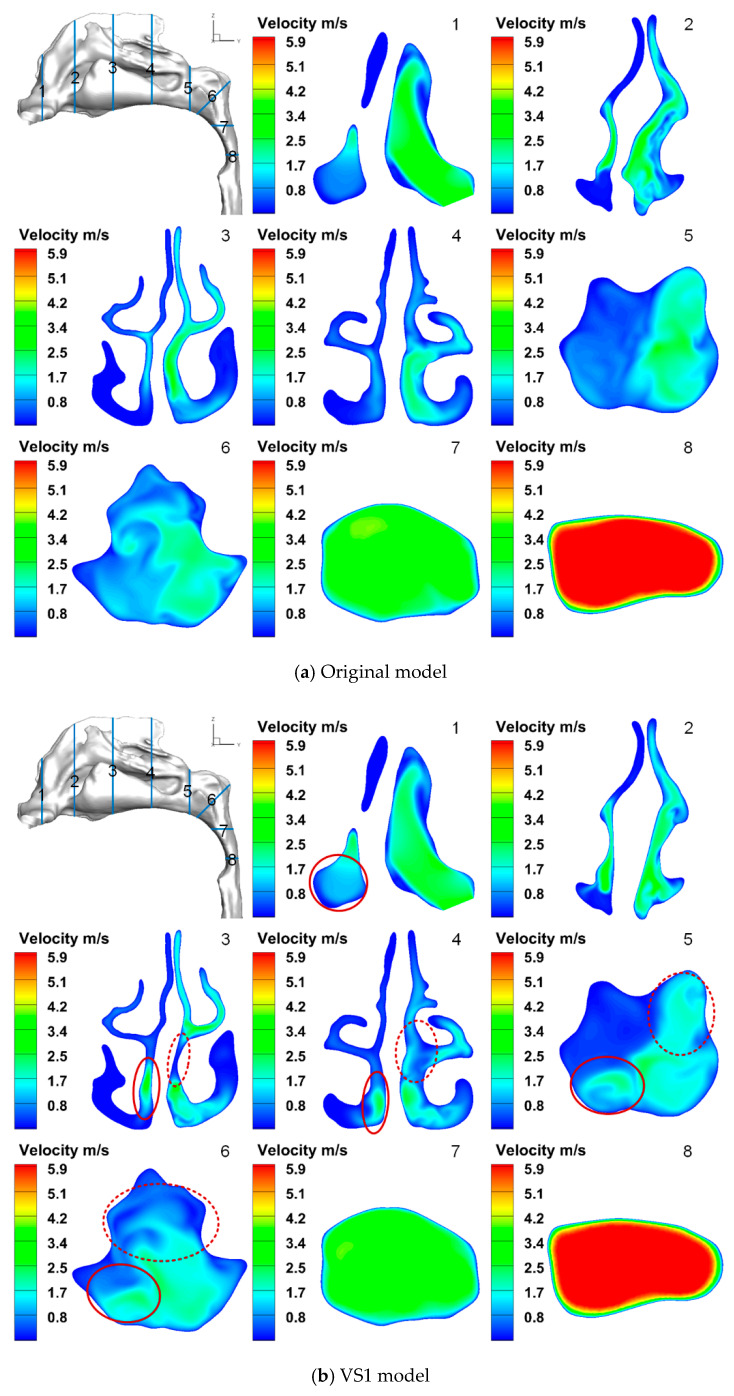

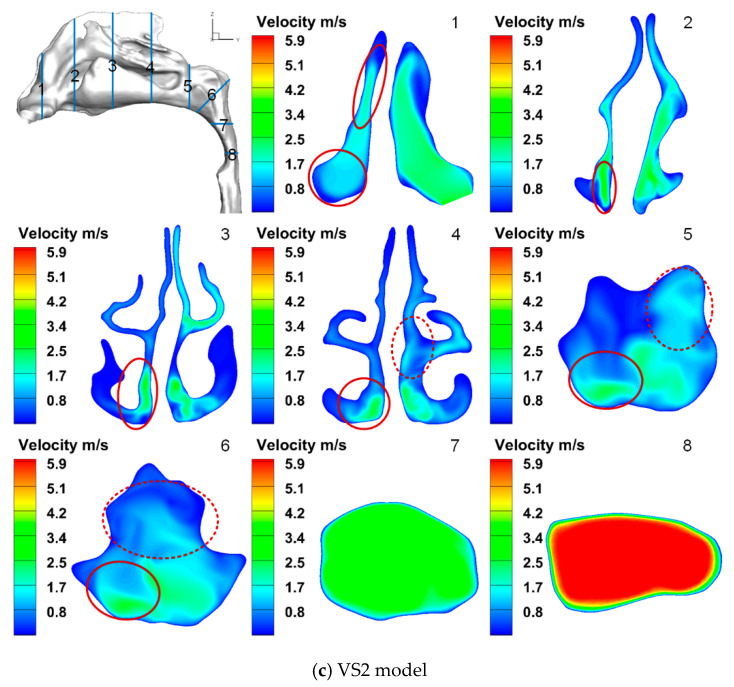
Velocity distributions of cross-sections of the nose-to-throat models at 15 L/min.

Figure 3 compares airflow velocity distributions in the cross sections among the three nose-to-throat models at *Q*_in_ = 15 L/min. For VS1 and VS2 models, the regions with increased (or decreased) velocity are circled out with solid (or dashed) lines. Because the velocity distributions of the three models at *Q*_in_ = 60 L/min are similar to the results shown in Figure 3, airflow velocity contour comparisons with *Q*_in_ = 60 L/min are not presented in this study. 

As shown in Figure 3a, it is evident that the septal deviation causes the uneven distribution of the airflow between two sides of the nasal cavity. The cross-sectional area of the left side is more significant than that of the right side from cross-section (CS) 1 to CS4. Accordingly, the air velocity of the left side is also higher than the right side from CS1 to CS4 in general. The airflow velocity in the middle and inferior meatus as well as close to the wall of the nasal septum on the left side, is higher than on the right side (see CS3 and CS4). When the two air streams merge in the nasopharynx, the velocity of the left half of the CS is approximately 1 to 2 m/s higher than the right half of the CS in the nasopharynx (see CS5). This leads to the strong secondary flow in the CS6, which moves the high-velocity region towards the center of the airway. Then the airflow gradually becomes uniform in CS7 and CS8.

VS1 model repairs the deviated septum, as shown in Figure 3b. The difference between the areas of the two sides becomes smaller in CS2 to CS4. The velocity in the lower part of the left side increases (red circle with solid line), while the middle part of the right side decreases (red circle with dash line). These changes also affect the velocity distributions in CS5 and CS6. The velocity in the upper left and lower right regions (upper right and lower left region shown in CS5 of Figure 3b) of the nasopharynx reduces and increases in CS5 compared to the prediction of the original model, respectively. It reduces the strength of the secondary flow shown in CS6.

VS2 model repairs the septal cartilage based on the VS1 model. The cross-sectional area of the right side of the nasal vestibule increases, as shown in CS1 of Figure 3c. Thus, the velocity of the right side increases in CS1 compared to the original model. The regions which have increased or decreased in velocity compared to the original model (red circles), expand based on the results of the VS1 model in CS1 to CS6. Therefore, the airflow allocation of the VS2 model improves compared with the VS1 model.

### 3.2. Particle Deposition Fractions (DFs)

**Figure 4:**
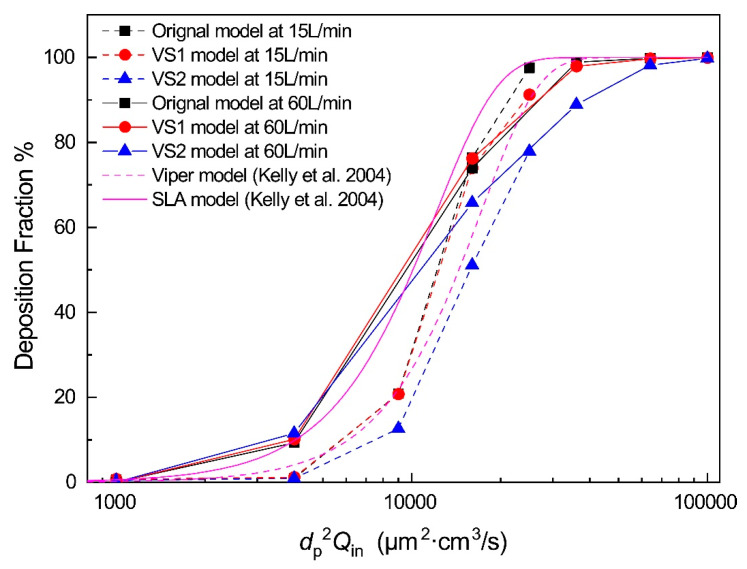
Relationship between deposition fraction of inhalable particles and particle diameter at *Q*_in_ = 15 L/min [19].

Figure 4 shows the relationship between the deposition fraction of inhalable particles and the impaction parameter [21] in the nasal airways with inhalation flow rates of 15 L/min and 60 L/min. The in vitro experimental measurements of a health subject [19] are also shown in Figure 4. The DF is defined as the fraction of the mass of particles deposited in the airway model to the mass of total inhaled particles.

For microparticles, the primary deposition mechanism in the upper airway is inertial impaction [19]. The trend of the deposition curves of the three models in this study are similar to the experimental results [19]. However, subject variability also can play an essential role in DFs [46]. Swift (1991) [47] and Guilmette et al. (1994) [48] reported similar curve shapes of the DF as functions of the impaction parameter (i.e., $d_{p}^{2}Q_{in}$), but with higher DF values.

Generally, it can be found that using Plan A (VS1 model), only DFs of large particles (8 and 10 μm) have limited reduction, while DFs of small particles (2-6 μm) are almost the same compared to the DFs of the original model. In contrast, Plan B (VS2 model) significantly decreases in DFs compared to the original model in the range of impaction para-meter $d_{p}^{2}Q_{in}$ between 9000 and 36,000 μm^2^·cm^3^/s. Specifically, the DFs of the VS2 model decreased by 8.24%, 25.32%, and 19.73% for 6, 8, and 10 μm particles at 15 L/min, respectively. For the turbulent flow, the DFs of the VS2 model decreased by 8.16% and 9.96% for 4 and 6 μm particles at 60 L/min, respectively. Thus, a better surgery plan for the deviated septum could also decrease particle deposition in the nasal cavity. This reduction in particle deposition can reduce the possibility of nasal diseases [49,50,51], as well as neurodegenerative and neurological disorders [52].

### 3.3. Localized Deposition Patterns

**Figure 5:**
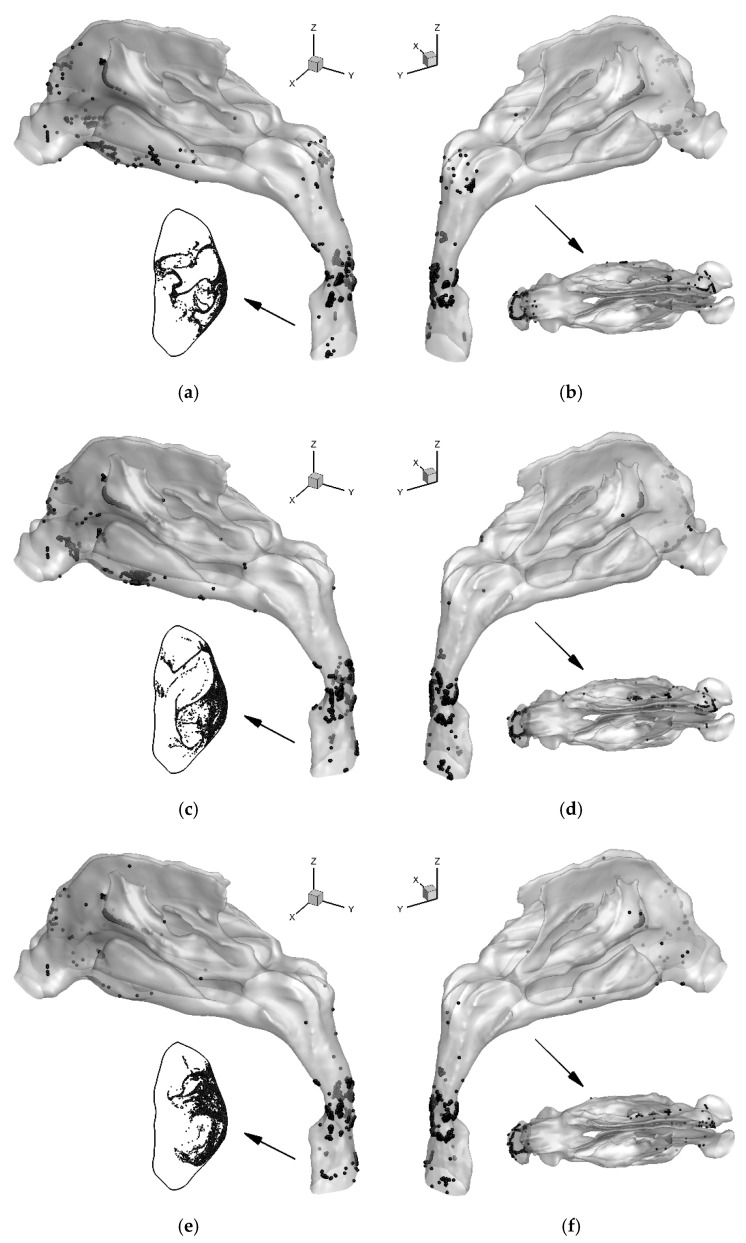
Final locations for the deposited and escaped 6 μm particles for the nose-to-throat models at *Q*_in_ = 15 L/min: (**a**) Left view of the deposition pattern and locations of escaped particles at the outlet for the original model, (**b**) Right and top views of the deposition pattern for the original model, (**c**) Left view of the deposition pattern and locations of escaped particles at the outlet for VS1 model, (**d**) Right and top views of the deposition pattern for VS1 model, (**e**) Left view of the deposition pattern and locations of escaped particles at the outlet for VS2 model, (**f**) Right and top views of the deposition pattern for VS2 model.

**Figure 6:**
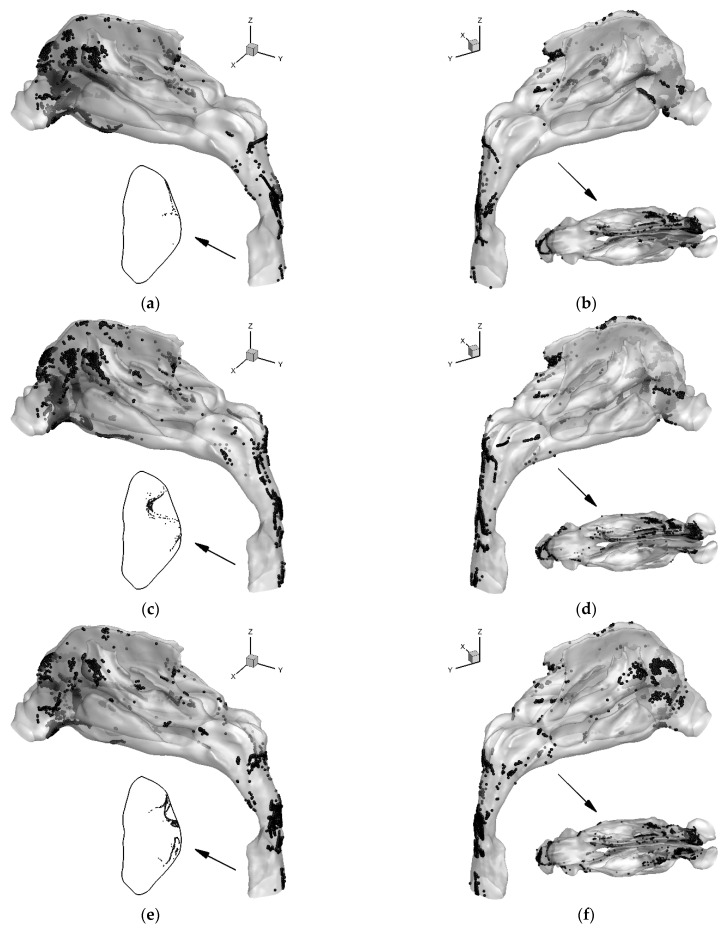
Final locations for the deposited and escaped 6 μm particles for the nose-to-throat models at *Q*_in_ = 60 L/min: (**a**) Left view of the deposition pattern and locations of escaped particles at the outlet for the original model, (**b**) Right and top views of the deposition pattern for the original model, (**c**) Left view of the deposition pattern and locations of escaped particles at the outlet for VS1 model, (**d**) Right and top views of the deposition pattern for VS1 model, (**e**) Left view of the deposition pattern and locations of escaped particles at the outlet for VS2 model, (**f**) Right and top views of the deposition pattern for VS2 model.

In order to analyze the effects of different virtual nasal septum deviation surgical protocols on the deposition of inhaled particles, the deposition patterns of 6 μm particles with *Q*_in_ = 15 L/min and 60 L/min were compared in Figure 5 and Figure 6, respectively. Regional deposition fractions of the nasal cavity, nasopharynx, laryngopharynx, and throat of the 6 μm particles are listed in Table 2.

Figure 5 illustrates the locations of the deposited and escaped 6 μm particles in the three nose-to-throat models at the inhalation flow rate of 15 L/min. The particle deposition mostly occurs in the nasal vestibule, laryngopharynx, and throat region. The nasal vestibule has a relatively small cross-sectional area, and therefore, the air velocity is higher in this region. The septal cartilage of this region has also deviated. Thus, the air streams from the two nostrils would change direction in the original model and VS1 model. The total DF in the VS1 model is similar to that in the original model at 15 L/min. However, the regional DFs in these two models are different, as shown in Table 2. The increase in regional particle deposition in the nasal cavity of VS1 model is located mostly on the nasal floor of the left side. As the high-velocity region of the airflow moves towards the lower part of the nasopharynx of the VS1 model (see CS5 in Figure 3b) compared to the original model, the number of particle deposition in the laryngopharynx and throat region is reduced. The VS2 model has larger cross-sectional area in the nasal vestibule because of the correction of the obstruction. The air and particle velocity in the nasal vestibule of the VS2 model decrease compared to the original model and VS1 model, which leads to the decrease in the regional DF of the nasal cavity in the VS2 model. Similar to the case of the VS1 model, the particle deposition in the laryngopharynx and throat region is also reduced, considering the reduction in the strength of the secondary flow (see CS6 in Figure 3c).

Figure 6 illustrates the locations of the deposited and escaped 6 μm particles in the three nose-to-throat models at the inhalation flow rate of 60 L/min. The number and area of the particle deposition increased compared to the results of 15 L/min shown in Figure 5 due to higher particle inertia and turbulent dispersion. For the original model, more particles deposit in the regions of the nasal valve and nasal vestibule. There is also more deposition near the upper part of the vestibule, which is close to the nasal bone and frontal sinus. The deposition increases on the left middle turbinate are also prominent. However, there is almost no particle deposited on the right middle turbinate. Compared to the DF under 15 L/min, the deposition in the laryngopharynx and throat is reduced because most particle deposition locates in the nasal cavity. The deposition in the nasopharynx increases due to the turbulent dispersion. The deposition pattern of VS1 is similar to the original model. However, the DF and deposition pattern of VS2 is different. Because the obstruction in the nasal vestibule is corrected, the air streams from the two nostrils become more parallel to the nasal cavity. This significantly reduces the particle deposition on the septal cartilage. The regional DF in the nasal cavity was reduced by 17% compared to that of the original model. For the right side of the nasal cavity, more air is inhaled compared to the original model and VS1 model. Thus, the inertia of the particle increases accordingly, which increases the deposition on the right side. However, the total DF of the VS2 model was only reduced by approximately 10% from that of the original model. Because the particles, which penetrate the nasal cavity, still deposit on the walls of the nasopharynx, laryngopharynx, and throat regions.

In general, hotpots of particle depositions locate at the entrance of the nasal cavity, the laryngopharynx, and throat regions, where the airway passages suddenly change directions causing strong inertial impaction. The observation is consistent with previous investigations of healthy individuals [53]. A better surgical plan reduces the particle deposition on the convex side, but slightly increases the deposition on the concave side. However, the overall deposition in the nasal cavity is reduced. Surgical results showed that nasal mucociliary transport rate improved after the surgery for nasal septal deviation [54]. This could result from the reduction in particle deposition in the nasal cavity, as shown in our predictions. Thus, the analysis of the computational particle-fluid dynamics assisted virtual surgery may benefit the patients with nasal airway obstruction more than the surgical outcome of better airflow distribution.

## 4. Conclusions

To analyze the reason for unsuccessful nasal septum surgery and seek optimal surgery plan, comparisons of airflow distribution and particle deposition patterns were performed among a pre-surgery patient-specific nose-to-throat model, as well as two post-surgery models via different surgical plans. Based on the CFD simulation results, the main conclusions are listed below:

- The CFD simulation combined with virtual surgery can help to evaluate the surgical plans for septum deviation and predict the airflow allocation between the two sides of the nasal cavity.
- Corrections of the obstruction in the nasal vestibule and caudal nasal septal deviation are important, beyond the correction of deviated deep areas of septal cartilage and bone. They can enhance the ratio of the inhalation flow rate of one side by up to 17%.
- The better virtual surgical plan for the septum deviation, i.e., the VS2 model in this study, not only improves the airflow distribution, but also significantly reduces particle deposition in the nasal cavity.
- For the septum deviation, the better virtual surgical plan reduces the particle deposition on the convex side, but slightly increases the deposition on the concave side. This may reduce the possibility of nasal diseases.

Potential future works may focus on the comparisons of heat and mass (water vapor) transfer between the original nasal airway and the ones after virtual surgeries. Besides the septal deviation, other nasal airway obstructions could also use the CFD simulation to analyze the outcome of the surgery. From the clinical aspect, more cases need to be accumulated before this method can be used in practice to select the best surgery plan for the patient. In vitro experiments should be carried out to validate the numerical simulations.

## Figures and Tables

**Table 1:** Flow rate distributions in the nasal cavities.

Nose-to-Throat Airway	Flow Rate (L/min)			Airflow Allocation (%)		Pressure Drop (Pa)
	Total	Left	Right	Left	Right	
Original model	15.0	12.6	2.4	84.0	16.0	31.6
	60.0	50.0	10.0	83.3	16.7	425.8
VS1 model	15.0	11.4	3.6	76.0	24.0	31.3
	60.0	46.3	13.7	77.2	22.8	416.2
VS2 model	15.0	10.0	5.0	66.7	33.3	30.9
	60.0	39.1	20.9	65.2	34.8	415.5

**Table 2:** Regional deposition fractions of 6 μm particles in the nose-to-throat models.

Flow Rate	Model	Regional Deposition Fraction (%)			Total Deposition Fraction (%)
		Nasal Cavity	Nasopharynx	Laryngopharynx and Throat	
15 L/min	Original	6.39	0.44	14.00	20.83
	VS1	9.67	0.06	10.98	20.71
	VS2	3.24	0.05	9.29	12.58
60 L/min	Original	89.78	2.50	6.59	98.87
	VS1	88.88	3.36	5.66	97.90
	VS2	62.53	7.11	19.28	88.92

## Data Availability

The data that support the findings of this study are available from the corresponding author upon reasonable request.

## Nomenclature

$C_{Dd}$	drag force coefficient for aerosol
$Cd_{\omega }$	cross-diffusion term
${\tilde{D}}_{k}$	modified term for destruction of turbulence kinetic energy
$D_{\omega }$	dissipation of *ω*
$d_{p}$	particle diameter
$\vec{g}$	gravitational acceleration
*k*	turbulence kinetic energy
*m_p_*	mass of the particle
$P_{k}$	production of turbulence kinetic energy
$P_{\gamma 1}$, $E_{\gamma 1}$	transition source terms
$P_{\gamma 2}$, $E_{\gamma 2}$	transition destruction terms
${\tilde{P}}_{k}$	modified term of $P_{k}$ with intermittency
$P_{\theta t}$	source term for transition momentum thickness Reynolds number
p	air pressure
${\tilde{Re}}_{\theta t}$	transport scalar for momentum thickness Reynolds number
t	time
u	fluid velocity
${\vec{u}}_{p}$	velocity vector of the particle
Greek	
γ	intermittency
μ	dynamic viscosity of the fluid
$\mu_{t}$	turbulent viscosity
υ	kinematic viscosity of the fluid
$\upsilon_{t}$	turbulent eddy viscosity
*ρ*	fluid density
$\sigma_{k}$	turbulent Prandtl number for *k*
$\sigma_{\omega }$	turbulent Prandtl number for *ω*
$\sigma_{\theta t}$	constant
*ω*	specific dissipation rate
